# Human lung adenocarcinoma cell cultures derived from malignant pleural effusions as model system to predict patients chemosensitivity

**DOI:** 10.1186/s12967-016-0816-x

**Published:** 2016-02-29

**Authors:** Giuseppe Roscilli, Claudia De Vitis, Fabiana Fosca Ferrara, Alessia Noto, Emanuela Cherubini, Alberto Ricci, Salvatore Mariotta, Enrico Giarnieri, Maria Rosaria Giovagnoli, Maria Rosaria Torrisi, Francesca Bergantino, Susan Costantini, Francesca Fenizia, Matilde Lambiase, Luigi Aurisicchio, Nicola Normanno, Gennaro Ciliberto, Rita Mancini

**Affiliations:** Department of Clinical and Molecular Medicine, Sapienza University of Rome, Rome, Italy; Takis srl, Rome, Italy; Laboratory of Research and Diagnostics, Department of Surgery “P.Valdoni”, Sapienza University of Rome, Rome, Italy; Azienda Ospedaliera S. Andrea, Rome, Italy; IRCCS Istituto Nazionale Tumori, Fondazione “G. Pascale”, Naples, Italy

**Keywords:** Malignant pleural effusions, NSCLC primary cultures, PDX, Next generation sequencing, In vitro chemosensitivity

## Abstract

**Background:**

Lung cancer is the leading cause of cancer related deaths and Malignant Pleural Effusion (MPE) is a frequent complication. Current therapies suffer from lack of efficacy in a great percentage of cases, especially when cancer is diagnosed at a late stage. Moreover patients’ responses vary and the outcome is unpredictable. Therefore, the identification of patients who will benefit most of chemotherapy treatment is important for accurate prognostication and better outcome. In this study, using malignant pleural effusions (MPE) from non-small cell lung cancer (NSCLC) patients, we established a collection of patient-derived Adenocarcinoma cultures which were characterized for their sensitivity to chemotherapeutic drugs used in the clinical practice.

**Methods:**

Tumor cells present in MPEs of patients with NSCLC were isolated by density gradient centrifugation, placed in culture and genotyped by next generation sequencing. In a subset of cases patient derived xenografts (PDX) were obtained upon tumor cell inoculation in rag2/IL2 knock-out mice. Isolated primary cultures were characterized and tested for drug sensitivity by in vitro proliferation assays. Additivity, antagonism or synergy for combinatorial treatments were determined by analysis with the Calcusyn software.

**Results:**

We have optimized isolation procedures and culture conditions to expand in vitro primary cultures from Malignant Pleural Effusions (MPEs) of patients affected by lung adenocarcinomas, the most frequent form of non small cell lung cancer. Using this approach we have been able to establish 16 primary cultures from MPEs. Cells were banked at low passages and were characterized for their mutational pattern by next generation sequencing for most common driver mutations in lung cancer. Moreover, amplified cultures were shown to engraft with high efficiency when injected in immunocompromised mice. Cancer cell sensitivity to drugs used in standard chemotherapy regimens was assessed either individually or in combination. Differential chemosensitivity and different mutation profiles were observed which suggests that this isolation method could provide a platform for predicting the efficacy of chemotherapy in the clinical setting. Most importantly for six patients it was possible to establish a correlation between drug response in vitro and response to therapy in the clinic.

**Conclusions:**

Results obtained using primary cultured cells from MPEs underscore the heterogeneity of NSCLC in advanced stage as indicated by drug response and mutation profile. Comparison of data obtained from in vitro assays with patients’ responses to therapy leads to the conclusion that this strategy may provide a potentially useful approach for evaluating individual chemosensitivity profile and tailor the therapy accordingly. Furthermore, combining MPE-derived primary cultures with their genomic testing allows to identify patients eligible to trials with novel targeted agents.

**Electronic supplementary material:**

The online version of this article (doi:10.1186/s12967-016-0816-x) contains supplementary material, which is available to authorized users.

## Background

Lung cancer is the leading cause of cancer-related death around the world. Non-small cell lung cancer (NSCLC) comprises about 80 % of all lung malignancies. More than half of NSCLC patients are diagnosed when tumor is at a late stage (III B and IV) and the only option is systemic chemotherapy [1, 2]. However, 5-year survival rate of these patients remains below 10 %. This low survival rate is due in large part to heterogeneity of tumor response to chemotherapy and lack of biomarkers or assays to guide the choice of the best chemotherapy. Ideally to be most effective therapy should be designed after careful assessment of the in vitro and/or in vivo chemosensitivity of patient’s tumor cells against a repertoire of potential therapeutic agents in order to select the best option for each patient on a personalized basis [3–6]. Furthermore, our increasing knowledge of the complex repertoire of actionable mutations occurring in genes driving uncontrolled tumor cell growth, combined with the development of efficient and low cost next generation sequencing technologies open up new opportunities for therapeutic intervention to be exploited in the future by genomic driven clinical trials [7].

Although pursued for many years, the translation of in vitro assay-informed therapy in clinical practice has been hampered by various technical problems, including the requirement of a high technical skill level, the large number of required tumor cells, and the long turnaround time [8, 9]. Consequently, treatment schedules and agents used in monotherapy or combination chemotherapy are still determined on the basis of the result of large clinical trials which do not take into account interpatient heterogeneity in response to chemodrugs. Hence, no reliable method has been developed to efficiently determine the best chemotherapy on an individualized basis.

The propagation of patients’ tumor cells is considered to be a source of material most closely related to the original tumor. In recent years great emphasis has been given to the possibility to transplant and propagate human tumors through serial passages in immunodeficient mice devoid of B, T and NK cells [10–12]. These patient derived xenografts (PDX) have been shown to be predictive of clinical outcome compared to conventional, cell line derived xenograft (CDX) models, in particular when therapeutic compounds were tested at clinically relevant doses (CRDs) [10, 13–18]. However, engraftment and tumor growth rates usually do not allow to assess in a timely manner patients’ chemosensitivity in order to instruct therapeutic decisions. PDX tumors are usually obtained from biopsies or resected primary tumors but this material is often of limited availability or cellular vitality, is not adequately preserved, and this accounts for the low engraftment rates observed in literature [19–21]. Moreover, tumor cells from sites other than the primary tumor, may increase our understanding of tumor evolution or tumor characteristics [22]. Therefore alternative sources of tumor cells are highly desirable in order to obtain sufficient material for chemosensitivity studies.

We have previously shown that tumor cells from pleural effusions of patients with NSCLC can be readily isolated and expanded in culture with high efficiency [23]. MPE is manifesting as a metastatic lesion and an advanced disease setting with poor prognosis. We showed that MPE-derived cell cultures achieve efficient tumor engraftment in recipient NOD/SCID mice, also upon inoculation of small number of cells, thus suggesting indirectly the presence of tumor initiating cells. Furthermore these cells were shown to be a promising system for the study of epithelial-to-mesenchimal transition (EMT) [24] and of the mechanisms responsible for resistance to EGFR inhibitors [25].

In the present study, we have analyzed a panel of low passages MPE-derived tumor cultures from NSCLC patients for a series of parameters including genetic alterations, rate of cell growth and chemosensitivity to chemotherapeutic agents currently used for the therapy of lung cancer in vitro. A subset of cultures was used to derive PDX models. Finally we tried to assess whether in vitro chemosensitivity to drugs may correlate with clinical responses. Our data, although limited to a small number of cases, suggest that MPE-derived tumor cultures could serve as a valuable tool for screening for sensitivity to chemotherapeutic agents and for genetic profiling useful to select the most effective therapy regimen.

## Methods

### Patients

A total of 16 patients (10 males and 6 females; mean age 72 + 7 years; clinical stage was determined according to the TNM classification) with histologically or cytologically confirmed diagnosis of adenocarcinoma of the lung complicated with malignant pleural effusion were studied. Therefore, all the patients were considered to be with a stage IV of disease. Tumor specimens were obtained for diagnosis or therapeutic indications. The study was approved by S. Andrea Hospital Ethics Committee 2010 (504/10) and all patients agreed to participate to the study signing an informed consent form.

### MPE primary cultures

Primary cultures were obtained as previously described [23]. Briefly, pleural fluids (200–1000 ml) were obtained by thoracentesis and collected aseptically in heparinized (10 U/ml) bottles/tubes. Samples were centrifuged at 300 g for 10 min, at 4 °C, and cell pellet was resuspended in 10 % FCS-RPMI (Invitrogen) or 1 % BSA/2 mM EDTA/PBS. Viability was determined by Trypan Blue exclusion dye and cell suspension was sedimented on Oncoquick (Greiner Bio One) gradient or Ficoll PLUS after the addition of RosetteSep cocktail (Stemcell). During the centrifugation step the cells were separated according to their different buoyant densities. The denser fluid components such as erythrocytes and leucocytes migrate into the lower phase through the bottom of the tube. The less dense cell fraction, including tumor cells, were enriched at the interphase layer formed between the plasma and the separation medium in the phase. After a harvesting and washing step, tumor cells were cultured in 10 % FCS-RPMI to obtain primary adherent cultures.

### Multiple gene mutation analysis by next generation sequencing

Tumor samples were analyzed with the Ion AmpliseqTM Colon and Lung Cancer Panel (Life Technologies) using Ion Torrent semiconductor sequencing as previously described [26].

### Whole exome sequencing

Next generation sequencing experiments, including quality control samples, were performed by Genomix4life S.R.L. (Baronissi, Salerno, Italy). Indexed libraries were prepared from 250 ng/ea DNA, after sharing with a Bioruptor sonicator, using the SureSelect Human All Exon kit (50 Mb; Agilent Technologies) according to the manufacturer’s instructions. Libraries were quantified using the Agilent 2100 Bioanalyzer (Agilent Technologies) and pooled in equimolar amounts to final concentration of 2 nM. Pooled samples were then subjected to cluster generation and sequencing using an Illumina HiSeq 2500 System (Illumina) in a 100 bp paired-end format at a final concentration of 8 pmol. The raw sequence files generated in fastq format underwent quality control analysis using FastQC (http://www.bioinformatics.babraham.ac.uk/projects/fastqc/).

The sequence reads were mapped against human genome (Homo sapiens Ensembl GRCh37, hg19) using Burrows-Wheeler Alignment (BWA version 0.7.7) software [27]. Sequence variations were detected by The Genome Analysis Toolkit (GATK; Broad Institute, Cambridge, MA, USA) software [28].

The Database for Annotation, Visualization and Integrated Discovery (DAVID; http://david.abcc.ncifcrf.gov) was used to perform functional annotation analysis of enriched gene ontology (GO) terms and KEGG pathways. Statistical significance was evaluated with a modified Fisher’s exact test (EASE score) and GO BIOCARTA and KEGG terms with P values ≤0.05 were considered significant.

### Chemicals and reagents

Cisplatin, carboplatin, docetaxel, vinorelbine, gemcitabine, gefitinib and erlotinib were obtained from SellekChem.

### Cytotoxicity assays

To determine effects on proliferation, MPE primary cultures were treated in triplicate with increasing concentrations of anticancer drugs for 72 h. Inhibition of cell proliferation was measured by colorimetric WST-1 assay. MPE primary cultures were plated in triplicate in 96-well flat-bottom plates at a density of 2000 cells/well and incubated overnight. Anticancer drugs were added at various concentrations in triplicates to the media and cells were cultured for an additional 72 h. At the end of the experiment, cell survival was determined by WST-1 assay (Roche) according to the manufacturer’s instructions. Absorbance was measured by spectrophotometer at a wavelength of 450 nm and viability was determined as percent of control cells (cells treated with the vehicle alone were defined as 100 % viable). Viability data were used to calculate EC50, concentration of agent at which the cell growth is inhibited by 50 %. EC50 values were determined by GraphPad (prism) and data points are presented as the average value ± the standard deviation (SD).

### Evaluation of drug combinations

To determine if the antitumor effects obtained with different drug combinations were synergistic, we calculated the combination index (CI) according to the Chou-Talalay method using Calcusyn software (Biosoft, Cambridge, UK). (CI> 1, antagonism; CI  =  1, additive effect; CI < 1, synergism). Since the Chou-Talalay model calls for cytotoxic agents to be used at a fixed dose ratio, we chose to use drug combination at equipotent ratio (ratio of EC50). Cells were treated with a combination that is four- to eightfold higher than the EC50, and used 1:3 serial dilutions of the highest concentration combination to generate the dose-response curve, in parallel each single agent in the combination was tested alone in the same manner. Cells were treated for 72 h and at the end of the experiment, cell survival was determined by WST-1 assay (Roche) according to the manufacturer’s instructions.

### Tumor engraftment studies

All studies have been performed in accordance with “Directive 2010/63/EU on the protection of Animals used for scientific purposes” and made effective in Italy by the Legislative Decree DLGS 26/2014. 6- to 8-weeks old Rag2/II2rg Double Knockout (Taconic) were utilized. After 1 week of acclimation they were housed five to a plastic cage and fed on basal diet (4RF24, Mucedola S.r.l.) with water ad libitum, in an animal facility controlled at a temperature of 23 ± 2 °C, 60 ± 5 % humidity, and with a 12 h light and dark cycle. All animal protocols used for this study were reviewed and approved by the Animal Welfare Body Takis/Plaisant. Animals were euthanized by cervical dislocation at the end of the study or when severe signs of suffering were observed. Before injection, cells were washed once in PBS and their pellet was resuspended in 50 % RGF matrigel (BD Biosciences) solution in Medium 199 and injected in the right flank of the mice in 200 µl volume/mouse. 1 × 106 or 5 × 106 cells/mouse were injected for a total of 4–5 mice/primary culture. Tumor growth was monitored weekly by caliper measurement and tumor volume was determined by the formula (D × d2)/2, where D was the longest diameter of the tumor. The tumor doubling time (DT) in days was estimated from the log linear tumor growth during the exponential phase (range, 100–1000 mm^3^).

## Results and Discussion

### Mutational analysis of MPE derived cultures reveals a high degree of heterogeneity

We established sixteen MPE-derived cultures, each one from sixteen different patients affected by adenocarcinoma (AdenoCa) of the lung. In order to minimize adaptation in cell culture that may lead to selection of cell subpopulations with particular growth advantage we tried to work in the majority of cases with early passage cultures from p2 to p7. Exceptions were PE d/10, PE e/10 and PE o/11 for which the earliest passage available for this study was p10. DNA was extracted as described in the methods section and subjected to Next Generation Sequencing (NGS) using the Ion AmpliseqTM Colon and Lung Cancer Panel (Life Technologies) targeting 500 hotspot regions in 22 known driver genes in lung cancer. The results reported in Table 1 show as expected a highly heterogeneous pattern of mutations which reproduces the known high degree of heterogeneity of AdenoCa of the lung [29].

**Table 1:** Mutations identified in MPE primary cultures

	KRAS	EGFR		PIK3CA	BRAF	MET	TP53	STK11	In vivo
		Exon19del	T790 M						
PE d/10 (p10)	46 % p.G12V (c. 35 G>T)								No
PE e/10 (p10)	87.9 % p.Q61H (c.183 A>C)					31.6 % p. T1010I (c. 3029 C>T)			Yes
PE b/11 (p3)					53.5 % p.V600E (c.1799 T>A)				Yes
PE g/11 (p6)									Yes
PE h/11 (p4)	43.1 % p.G12D (c. 35 G>A)								No
PE i/11 (p5)	37.2 % p.Q61 K (c.181 C>A)						100 % p.R175H (c.524 G>A)		Yes
PE n/11 (p3)									Yes
PE o/11 (p10)	98.8 % p.Q61H (c.183 A>T)			54.2 % p.E545 K (c.1633 G>A)				100 % p.Q37* (c.109 C>T)	No
PE p/11 (p7)		100 % p.E746_A750del (c.2235_2249del15)	17.2 % p.T790 M (c.2369 C>T)				100 % p.R248Q (c.743 G>A)		No
PE r/11 (p5)		47 % p.E746_A750del (c.2235_2249del15)							No
PE s/11 (p3)		42 % p.E746_A750del (c.2235_2249del15)	15 % p.T790 M (c.2369 C>T)				14.3 % p.S241F (c.722 C>T)		Yes
PE u/11 (p6)		49 % p.E746_A750del (c.2235_2249del15)	11.3 % p.T790 M (c.2369 C>T)						Yes
PE v/11 (p2)		57 % p.E746_A750del (c.2235_2249del15)							Yes
PE z/11 (p4)									Yes
PE b/12 (p4)	100 % p.Q61H (c.183 A>C)			49.1 % p.E545 K (c.1633 G>A)				100 % p.Q37* (c.109 C>T)	Yes
PE f/13 (p3)									No

* nonsense mutation

Interestingly, in our small sample we observed in several cases a mutation frequency similar to that reported by TCGA, namely KRAS 37.5 % vs 32.2; BRAF mutations 6 vs 6 %; MET 6 vs 4.3 %. TP53 mutations were unexpectedly low (18.75 vs 40 %). KRAS and EGFR mutations never occurred in the same samples. Activating EGFR mutations were detected in 31 % of cases vs 11.3 % in TCGA. This is in line with previous reports that showed an increased frequency of EGFR mutation in malignant pleural effusions vs tumor tissue [30]. Importantly in 3 out of 5 cases of activating EGFR mutations (all Exon 19 deletions) we observed the simultaneous presence of the gatekeeper T790 M mutation which confers resistance to TKIs although none of these patients was previously treated with EGFR TKIs. The presence of a pre-existing gatekeeper T790 M mutation together with activating EGFR mutations has already been reported at variable frequency depending upon the DNA sequencing methodology and has been shown to affect time to disease progression after TKI therapy when mutation frequency is above 3 % as in our cases (see Table 1) [31, 32]. Finally, we also observed a high rate (12.5 %) of mutations in the tumor suppressor STK11 gene.

In several cases mutations were detected at low frequencies (between 10 and 20 % and in any case significantly below 50 %), which suggests the existence of a heterogeneous populations of cells. This has been reported before in colorectal cancer [33, 34], breast cancer [35], and lung cancer [36, 37].

Overall, using this small panel of 22 genes it was possible to identify actionable mutations in driver genes in a large proportion of cases. This may provide new therapeutic options for this type of patients if this information is used to conduct genomic driven trials with new targeted agents.

**Fig. 1:**
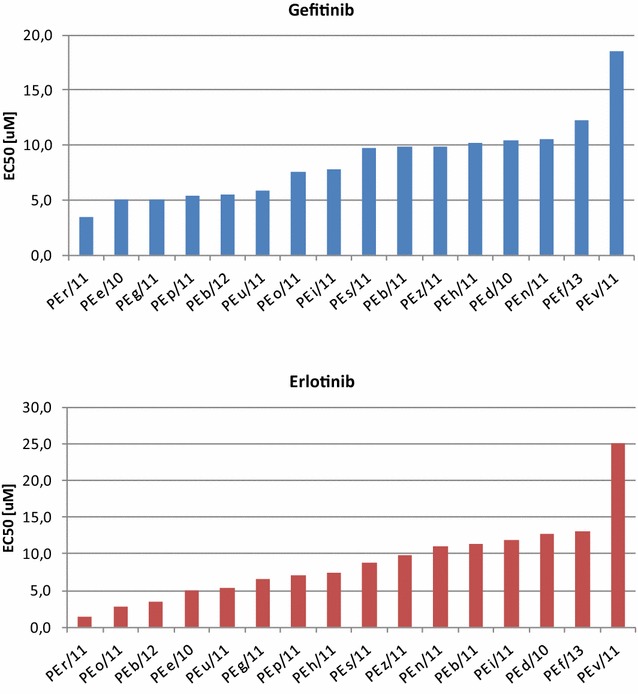
Sensitivity of primary cultured MPE tumor cells to gefitinib and erlotinib

We also determined the EC50 values for EGFR TKIs gefitinib and erlotinib for all 16 cell cultures (Fig. 1). The relative pattern of sensitivity was similar but not identical. The vast majority of cultures showed an intermediate to high degree of resistance to these drugs, also in the presence of EGFR sensitizing mutations, with the only exception of r/11 (one of the two cultures bearing the presence of the Exon19 deletion in EGFR without the coexistence of the resistance mutation T790 M) which showed a good sensitivity to both gefitinib and erlotinib. However, the other culture with the same NGS mutational pattern, namely v/11 was highly resistant to both drugs, which suggests that chemosensitivity may be affected by other mutational or epigenetic changes not revealed by this analysis. The three cultures with coexisting Exon19 deletion and T790 M mutations, p/11, s/11 and u/11, were as expected intermediate to highly resistant to both drugs. Overall however, the NGS mutational pattern reported Table 1 did not allow to predict chemosensitivity to both gefitinib and erlotinib shown in Fig. 1. This was also the case for the six cultures bearing KRAS mutations (d/10, e/10, h/11, i/11, o/11 and b/12) alone or in combination with others. Although it is interesting to observe that within this group the more sensitive cultures were those bearing mutations in PIK3CA, their response to drugs spanned the entire range.

In conclusion, although we are cognizant of the small number of cases analyzed, in our system drug sensitivity to TKIs could not be entirely anticipated by NGS data. This supports two important concepts: (a) sensitivity to TKIs may be strongly affected by additional genetic or epigenetic changes not revealed by our present NGS panel; (b) empirical determination of drug sensitivity is an added value to predict drug sensitivity in the clinic.

### Whole exome analysis reveals a pattern of mutations affecting distinct signaling and metabolic pathways

To identify the metabolic pathways affected in MPE-derived cultures, we performed whole exome sequencing on five randomly chosen samples, b/12, n/11, o/11, s/11 and u/11 which taken together well represent the differential degree of sensitivity to gefitinib and erlotinib. This analysis generated an average number of mappable sequence data equal to 55.19 ± 21.40 Gb (Additional file 1: Table S1). A percentage higher than 99.5 % of the total reads produced was mapped to the reference human coding exome (Homo sapiens Ensembl GRCh37, hg19). From this amount of data, GATK version 3.3-3 identified an average of 219,178 ± 57,043 genetic variants, among which 21,220 ± 349 exomic and splice site type and 9830 ± 159 non-synonymous variants. Focusing on two samples having the same mutated genes by targeted resequencing (see previous paragraph), b/12 and o/11, it is possible to underline that they have a total number of variants equal to 20,963 and 21,499 among which 10,214 and 10,499 non-synonymous variants respectively. Then we evaluated also the total number of shared non-synonymous variants among the five MPE-derived cultures that resulted to be 4060 in 2573 different genes. The common mutations were distributed through the whole genome but higher frequency were found in chromosomes 1, 11 and 19 (Additional file 2: Figure S1)

To investigate the possible biological and metabolic role of the mutated genes, we categorized them into enriched categories according to GO molecular function classification and BIOCARTA and KEGG pathway analysis.

The top GO categories that resulted significantly enriched with a P value ≤0.05 and that comprised a number of genes higher than 50 were: ion binding, calcium ion binding, peptidase activity, olfactory receptor activity, structural molecule activity, cytoskeletal protein binding, endopeptidase activity, carbohydrate binding and actin binding.

**Fig. 2:**
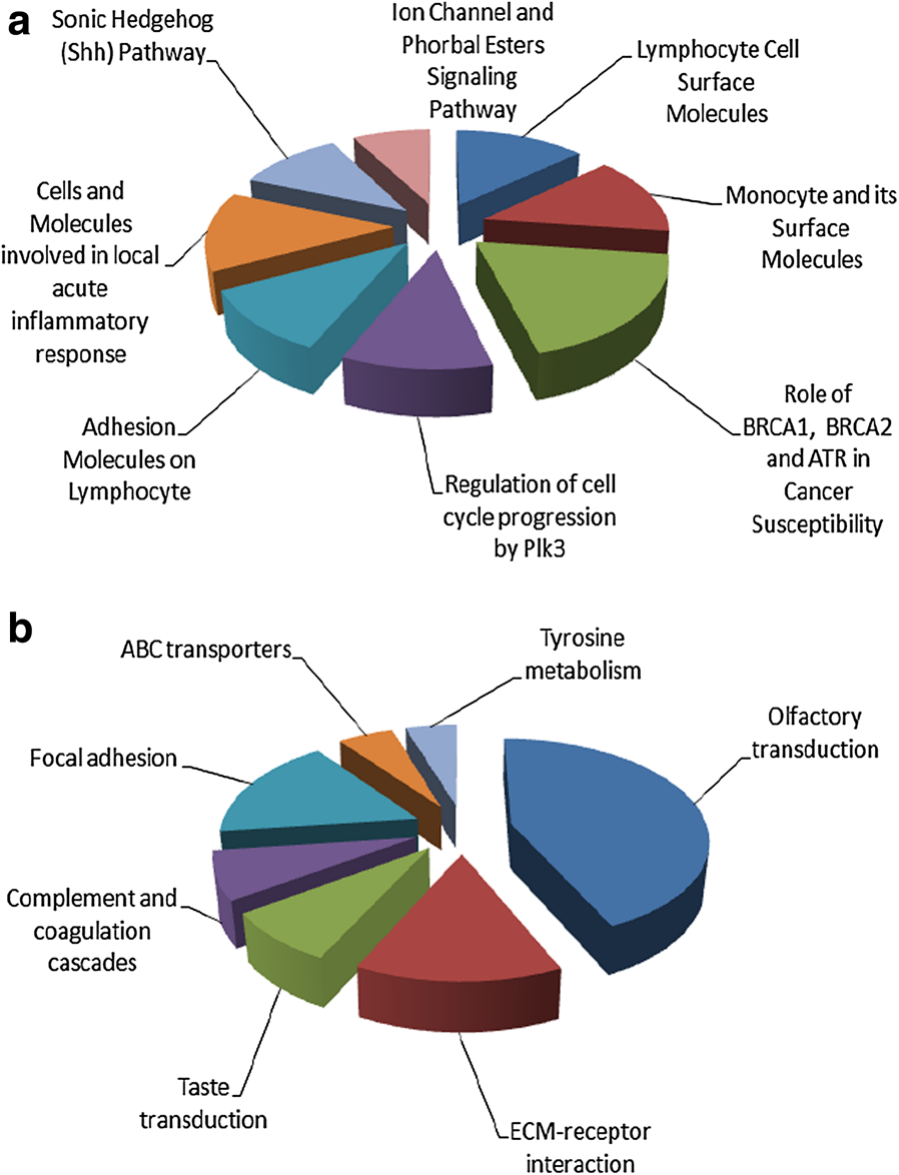
The 2573 mutated genes were categorized into enriched categories according to BIOCARTA (**a**) and KEGG (**b**) pathway analysis

Moreover, to evaluate the enrichment in signaling pathways, we performed BIOCARTA and KEGG analysis on the 2573 genes. The top 6 enriched pathways in BIOCARTA database were: B Lymphocyte Cell Surface Molecules, Monocyte and its Surface Molecules, Role of BRCA1, BRCA2 and ATR in Cancer Susceptibility, Regulation of cell cycle progression by Plk3, Adhesion Molecules on Lymphocyte, and Cells and Molecules involved in local acute inflammatory response (Fig. 2a). Among the genes involved in these pathways there are CD44, some integrins, BRCA1, BRCA2 and TP53. The top 7 enriched pathways in KEGG database were: Olfactory transduction, ECM-receptor interaction, Taste transduction, Complement and coagulation cascades, Focal adhesion, ABC transporters, and Tyrosine metabolism (Fig. 2b). In these different pathways we found involved the olfactory receptor family, the taste receptors, some coagulation and complement factors, a2-microglobulin, ATP-binding cassette family, alcohol dehydrogenases, collagen type family, integrins, laminins, ERBB2, protein kinase C and cyclin D3.

### In vivo tumor growth often leads to selection of cell populations with a different mutational pattern

Malignant pleural effusion is a poor prognostic factor for patients with lung cancer and the treatment is merely palliative [38]. Patient-derived tumor xenograft (PDX) models have been established and increasingly used for preclinical studies of targeted therapies in recent years. However, PDX mouse models are difficult to obtain with low percentage of success [19] and in particular, patient-derived non-small cell lung cancer (NSCLC) xenograft are relatively few in number and are limited in their degree of genetic characterization. We have characterized the isolated primary cultures for their ability to establish tumor xenograft in rag2-/Il2- double knock-out mice. Among the primary culture tested, more than 80 % of them were able to grow when injected s.c. establish subcutaneous xenografts. Moreover a great variability in latency time and doubling time was observed as shown in (Additional file 1: Table S2).

We extracted gDNA from tumors grown in PDX and carried out mutational analysis with the same NGS panel used for MPE-derived cells grown in vitro and the comparison of the pattern of mutations in the same samples between in vitro and in vivo is shown in Table 2.

**Table 2:** Comparison of Mutations identified in MPE primary cultures and PDX

	KRAS	EGFR		PIK3CA	BRAF	MET	TP53	STK11
		Exon19del	T790 M					
PE e/10 (p10)	87.9 % p.Q61H (c.183 A>C)					31.6 % p. T1010I (c. 3029 C>T)		
PE e/10 (PDX)	50.4 % p.Q61H (c.183 A>C)			49.2 % p.E545 K (c.1633 G>A)				44.5 % p.Q37* (c.109 C>T)
PE b/11 (p3)					53.5 % p.V600E (c.1799 T>A)			
PE b/11 (PDX)		100 % p.E746_A750del (c.2235_2249del15)	15.2 % p.T790 M (c.2369 C>T)				97.6 % p.R248Q (c.743 G>A)	
PE g/11 (p6)								
PE g/11 (PDX)	75.5 % p.Q61H (c.183 A>C)			49.5 % p.E545 K (c.1633 G>A)				77.7 % p.Q37* (c.109 C>T)
PE i/11 (p5)	37.2 % p.Q61 K (c.181 C>A)						100 % p.R175H (c.524 G>A)	
PE i/11 (PDX)	29.4 % p.Q61 K (c.181 C>A)						100 % p.R175H (c.524 G>A)	
PE n/11 (p3)								
PE n/11 (PDX)	27 % p.Q61 K (c.181 C>A)						100 % p.R175H (c.524 G>A)	
PE s/11 (p3)		42 % p.E746_A750del (c.2235_2249del15)	15 % p.T790 M (c.2369 C>T)				14.3 % p.S241F (c.722 C>T)	
PE s/11 (PDX)		100 % p.E746_A750del (c.2235_2249del15)	15.8 % p.T790 M (c.2369 C>T)				94.7 % p.R248Q (c.743 G>A)	
PE u/11 (p6)		49 % p.E746_A750del (c.2235_2249del15)	11.3 % p.T790 M (c.2369 C>T)					
PE u/11 (PDX)		92 % p.E746_A750del (c.2235_2249del15)	11.8 % p.T790 M (c.2369 C>T)				100 % p.R248Q (c.743 G>A)	
PE v/11 (p2)		57 % p.E746_A750del (c.2235_2249del15)						
PE v/11 (PDX)		87.5 % p.E746_A750del (c.2235_2249del15)					93.5 % p.R248Q (c.743 G>A)	
PE z/11 (p4)								
PE z/11 (PDX)		100 % p.E746_A750del (c.2235_2249del15)	14.2 % p.T790 M (c.2369 C>T)				98.8 % p.R248Q (c.743 G>A)	
PE b/12 (p4)	100 % p.Q61H (c.183 A>C)			49.1 % p.E545 K (c.1633 G>A)				100 % p.Q37* (c.109 C>T)
PE b/12 (PDX)	65.7 % p.Q61H (c.183 A>C)			49 % p.E545 K (c.1633 G>A)				63 % p.Q37* (c.109 C>T)

* nonsense mutation

It can immediately be appreciated that in vivo growth causes an overall increase in the frequency of mutations. Out of the 10 samples analyzed in parallel, only 3 (b/12, s/11 and i/11) maintain the same mutational pattern, with some differences in the abundance of cells with mutations between in vitro culture and PDX. In the remaining samples the pattern of mutations is dramatically different. In particular we can observe three major phenomena: (a) dramatic increase of TP53 mutations which now slightly exceed the frequency observed in TCGA (50 vs 40 %), (b) appearance of mutations in MPE-derived samples where no mutation had been detected in vitro (g/11, n/11), (c) disappearance of mutations present in the in vitro culture at low frequency and appearance of new mutations very often at high frequency.

This finding is of relevance because it suggests that some primary cultures are intrinsically highly heterogeneous in their composition and contain at very low percentages subclones carrying mutations undetectable by NGS. A dynamic adaptation takes place in the local tumor microenvironment by which these subclones emerge, while others disappear. Based on these data we believe that combining genetic testing of both primary cultures and PDX-derived tumors may provide a more complete analysis of the spectrum of tumor mutations for this group of patients.

### In vitro drug testing shows a high degree of differences in drug sensitivity in MPE-derived cultures

**Fig. 3:**
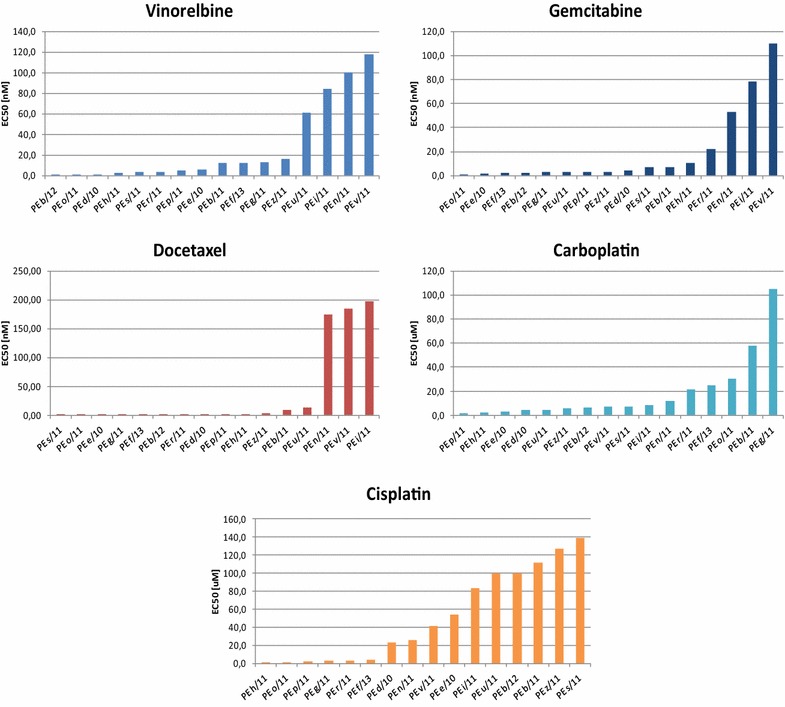
Sensitivity of primary cultured MPE tumor cells to chemotherapeutic drugs

MPE-derived primary cultures were analyzed for their sensitivity to different chemotherapeutic drugs used in conventional adenocarcinoma lung therapies, cisplatin, carboplatin, gemcitabine, vinolrebine and docetaxel. Chemodrugs were dosed as single agents and results showed a great variability in the response obtained (Fig. 3). Among the primary cultures PE v/11, i/11, n/11 displayed cross resistance between most of the drugs tested. The majority of primary cultures was resistant to cisplatin (10 out of 16 of them showed an EC50 higher than 20 μM), while we observed a greater degree of sensitivity to carboplatin (only 5 out of 18 had an EC50 higher than 20 μM). Carboplatin is a second generation platinum based compound with a different toxicity profile than cisplatin. Apart from the toxicity profile, the two compounds have a very similar mechanism of action, inducing the same type of DNA-platinum adducts, and in terms of acquired resistance, usually show cross resistance [39–41]. Reduced sensitivity to cisplatin may reside in an active drug efflux, like Copper efflux systems [42, 43], that preferentially inhibits drug accumulation in the cells. Since several mechanisms of resistance to platinum-based compounds have been described [44], a final explanation cannot be provided without the execution of further studies.

**Fig. 4:**
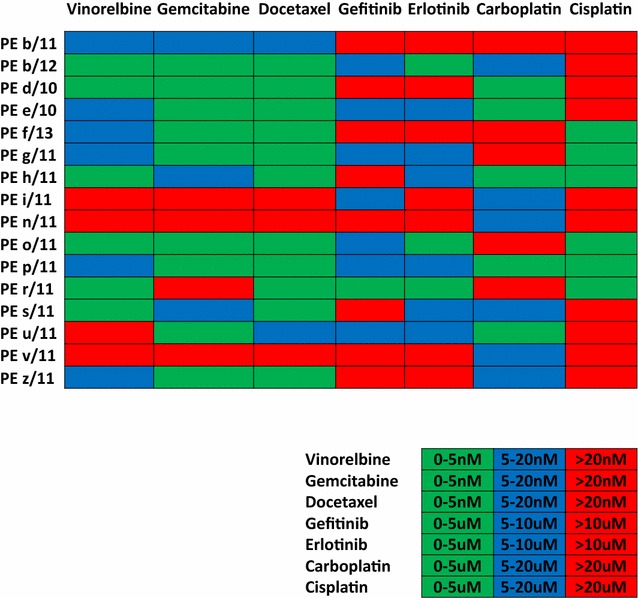
Sensitivity heat map of PE primary cultures treated with single chemotherapeutic agent

In Fig. 4 is a heat map of single drug sensitivity to the five chemodrugs and to the two TKIs. This highlights that the two drugs for which there is a higher degree of sensitivity are gemcitabine and docetaxel. This is intriguing because these are usually not the first line treatment for this group of patients. Our findings therefore underscore the importance of this type of in vitro chemosensitivity studies to better instruct clinicians about the most efficacious therapy to use for this highly aggressive manifestation of disease.

### In vitro assays lead to the identification of more effective synergistic combinations

**Fig. 5:**
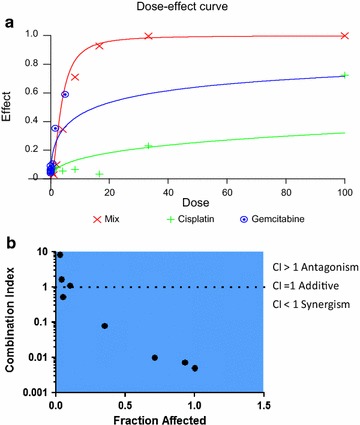
In vitro evaluation of cisplatin and gemcitabine co-treatment and determination of Combination Index (CI) for PE r/11. **a** Dose-Effect curve and **b** Combination Index plot obtained by CalcuSyn software

Patients affected by NSCLC are usually treated with combination of drugs especially when diagnosed at late stage when there are no other treatment options [45, 46]. MPE-derived cultures were assessed for their sensitivity to combination of drugs mostly used in the clinical settings and the results were evaluated by the Chou-Talalay method [47]. In Fig. 5a, b is shown as example the case of the MPE-derived r/11, where a strong synergism between cisplatin and gemcitabine could be observed. We conducted a more systematic analysis of the combinatorial effect of vinolrebine, docetaxel and gemcitabine in 6 samples, g/11, n/11, p/11, r/11, s/11 and u/11 and the results are reported in Table 3. This analysis allowed to distinguish three distinct cases: additivity as in the case of g/11, antagonism in n/11 and p/11 and finally synergism in r/11, s/11 and u/11.

**Table 3:** In vitro determination of Combination Index (CI) of cisplatin combined with gemcitabine, or vinorelbine or taxotere

MPE culture	Cisplatin combination	CI
PE g/11	Vinorelbine	1
	Gemcitabine	1
	Docetaxel	1
PE n/11	Vinorelbine	>1
	Gemcitabine	>1
	Docetaxel	>1
PE p/11	Vinorelbine	>1
	Gemcitabine	>1
	Docetaxel	<1
PE r/11	Vinorelbine	<1
	Gemcitabine	<1
	Docetaxel	<1
PE s/11	Vinorelbine	<1
	Gemcitabine	<1
	Docetaxel	<1
PE u/11	Vinorelbine	<1
	Gemcitabine	<1
	Docetaxel	1

Therefore, we can conclude that MPE-derived cultures can be used to determine not only the degree of chemosensitivity to single drugs but also the degree of synergism to combinations of different chemotherapeutic agents.

### Correlation between in vitro drug sensitivity and in vivo response to therapy

For six MPE-derived cultures for which we were able to assess in vitro chemosensitivity we had information about the first line therapy adopted and the clinical response. These data are shown in Table 4.

**Table 4:** Available patient data on response to drug treatment

Patient	Primary culture	1st line therapy	1st line response (3 cycle)	1st line response (6 cycle)	2nd line therapy	2nd line response (3 cycle)
#1	PE g/11	Cis + Vin	PR	SD	Erlotinib	PD
#2	PE n/11	Cis + Vin	PD			
#3	PE p/11	Cis + Gem	SD	SD	Pemetrexed	PD
#4	PE r/11	Cis + Gem + Beva	PR			
#5	PE s/11	Cis + Taxotere	PR			
#6	PE u/11	Cis + Gem + Beva	PR			

Clinical response: *PD* progression disease, *SD* stable disease, *PR* partial regression, *CR* complete

Two patients #1 and #2 from whom the g/11 and n/11 cultures were derived respectively, were treated with the combination of cisplatin and vinolrebine. Patient #3 from whom p/11 was obtained, was treated with the combination of cisplatin and gemcitabine. Patients #4 and #6 from whom r/11 and u/11 were obtained were treated with the combination cisplatin + emcitabine +Bevacizumab. Finally patient #5 from whom s/11 was obtained was treated with the combination of cisplatin plus Taxotere.

Patients, #1, #4, #5 and #6 developed a partial response. This nicely correlated with the in vitro sensitivity of the corresponding cultures to at least one of the drug used in single treatments, i.e., g/11 (patient #1) to cisplatin; r/11 (patient #4) to cisplatin; s/11 (patient #5) to docetaxel, u/11 (patient #6) to gemcitabine. Stable disease was observed in patient #3 where the corresponding p/11 culture showed in vitro sensitivity to both agents used in therapy cisplatin and gemcitabine. Finally, patient #2 underwent progressive disease upon treatment with cisplatin plus vinolrebine in line with the in vitro resistance of the corresponding n/11 culture to both agents.

Although the number of the patients enrolled is low, it is intriguing to observe that in vitro chemosensitivity data match with clinical responses to therapy, which suggests that the use of MPE-derived cultures may be helpful to predict in the future the best treatment for NSCLC patients with malignant pleural effusions. To confirm the significance of the acquired data a larger study with the appropriate number of patients would be required.

## Conclusions

Malignant pleural effusion (MPE) is an unfavorable complication of NSCLC. MPE severely restricts quality of life and has a poor prognosis. It is a metastatic manifestation of the disease caused by a combination of different processes such as inflammation, enhanced angiogenesis and vascular leakage. Response to therapies is usually poor because of relatively high tumor burden and chemoresistance. This last feature is linked to the presence of Cancer Stem Cells as we and others have shown in previous studies [23, 48, 49]. We believe that such an aggressive disease requires a novel therapeutic strategy based on a personalized approach and that this should stem from the combination of genetic analysis of tumor cells as well as from accurate prediction of chemosensitivity. In this study we have demonstrated the possibility to expand an initial population of MPE-derived tumor cells both through short term primary cultures as well as xenografts in order to carry out chemosensitivity assay and genetic characterization for most commonly altered driver genes in NSCLC. Data obtained highlight the extreme genetic heterogeneity of this disease but also the possibility to identify in the majority of cases mutations in actionable genes. Therefore it should be possible to link this type of genetic characterization to patients’ enrollment in umbrella clinical trials with novel targeted agents [50]. Limiting our analysis to more conventional chemotherapies, a more feasible and immediate application of our findings stems from the analysis of in vitro chemosensitivity of MPE-derived cultures. We show here that sensitivity to chemotherapeutic agents is highly heterogeneous. However we also show that it is possible in all cases to identify a combination of drugs that have a synergistic effect in inhibiting tumor growth and that this combination is often not the most frequent combination used in the clinic. Finally, although limited to a very small number of cases we show that in vitro chemosensitivity data match with patients’ response to therapy in the clinic. We believe that streamlining MPE sample processing with in vitro chemosensitivity and genetic analysis by NGS could open up new therapeutic options for this group of patients currently with limited therapeutic options and short term life expectancy. However one has to take into account that MPEs are present only in a small subset of the patients with advanced lung cancer, are a negative prognostic factor and strongly correlate to short patients’ survival. Thus, their clinical use could be limited by the extreme cancer burden and by the short life expectancy of patients. Therefore it would be of much greater clinical impact to translate our approach to primary lung tumors and to expand in vitro and/or in vivo the usually very small amount of material obtained from biopsies in order to be able to fully assess chemosensitivity.

## Additional files

10.1186/s12967-016-0816-x Whole genome exome sequencing. Analysis of sequence reads. **Table S2.** Doubling time and latency of MPE primary cultures in Rag2/Il2rgamma double knock-out mice.
10.1186/s12967-016-0816-x Distribution in the chromosomes of the common non-synonymous variants.

## Abbreviations

MPE: malignant pleural effusion
NSCLC: non-small cell lung cancer
PDX: patient derived xenograft
CDX: cell line derived xenograft
CRD: clinically relevant doses
EMT: epithelial-to-mesenchimal transition
CI: combination index
DT: doubling time
PD: progression disease
SD: stable disease
PR: partial regression
CR: complete regression
