# Supplementary material for: Human lung adenocarcinoma cell cultures derived from malignant pleural effusions as model system to predict patients chemosensitivity
Source: J Transl Med. 2016 Feb 29;14:61. doi: 10.1186/s12967-016-0816-x (PMC4772534; doi:10.1186/s12967-016-0816-x)
Supplement: Supplementary file 1 — 10.1186/s12967-016-0816-x Whole genome exome sequencing. Analysis of sequence reads. Table S2. Doubling time and latency of MPE primary cultures in Rag2/Il2rgamma double knock-out mice. [file 12967_2016_816_MOESM1_ESM.docx]

Table S1.Whole genome exome sequencing. Analysis of sequence reads.

|  | **PE/b12** | **PE/n11** | **PE/o11** | **PE/s11** | **PE/u11** |
| --- | --- | --- | --- | --- | --- |
| **Number of mappable reads** | 42860262 | 43759428 | 35610478 | 66199357 | 87510472 |
| **% mapped reads** | 99,6 | 99,6 | 99,6 | 99,7 | 99,7 |
| **Total of identified variants** | 178303 | 188871 | 167760 | 268925 | 292033 |
| **Exonic and splice site (SS) variants** | 20963 | 21469 | 21499 | 20734 | 21433 |
| **Non-synonymous variants** | 9701 | 9899 | 9954 | 9621 | 9976 |
| **Unknown SNP variants** | 470 | 483 | 499 | 476 | 514 |

Table S2. Doubling time and latency of MPE primary cultures in Rag2/Il2rgamma double knock-out mice.

|  | **In vivo** | | **In vitro** |
| --- | --- | --- | --- |
|  | **DT**  **(days)** | **Latency (days)** | **DT**  **(days)** |
| **PE g/11** | 10.4 | 22 | 2.1 |
| **PE b/12** | 10.3 | 23 | 2.2 |
| **PE i/11** | 15 | 28 | 3.3 |
| **PE o/11** | 6.7 | 30 | 1.9 |
| **PE s/11** | 17 | 30 | 1.2 |
| **PE z/11** | 17 | 37 | 1.8 |
| **PE e/10** | 8.4 | 42 | 1.2 |
| **PE v/11** | 43 | 44 | 1.4 |
| **PE n/11** | 5 | 50 | 3.8 |
| **PE u/11** | 17 | 72 | 2.2 |
| **PE f/13** | 19 | 118 | 2.4 |
| **PE h/11** | - | - | 3.1 |
| **PE d/10** | - | - | 3.2 |
| **Average** | **15.3** | **45.1** | **2.3** |

DT: Doubling time
