# Supplementary figures and images for: Human lung adenocarcinoma cell cultures derived from malignant pleural effusions as model system to predict patients chemosensitivity
Source: J Transl Med. 2016 Feb 29;14:61. doi: 10.1186/s12967-016-0816-x (PMC4772534; doi:10.1186/s12967-016-0816-x)

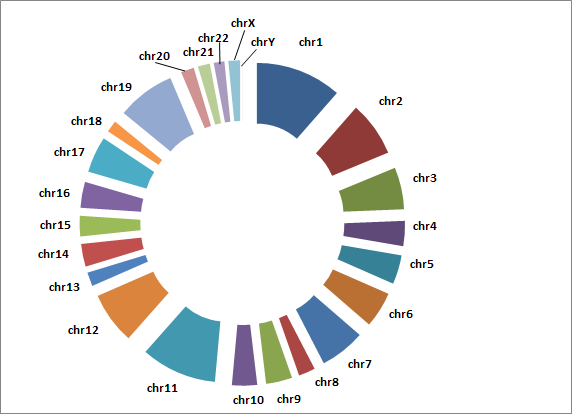


Figure S1. Distribution in the chromosomes of the common non-synonymous variants

Supplement: Supplementary file 2 — 10.1186/s12967-016-0816-x Distribution in the chromosomes of the common non-synonymous variants. [file 12967_2016_816_MOESM2_ESM.docx]
